# The representation of priors and decisions in the human parietal cortex

**DOI:** 10.1371/journal.pbio.3002383

**Published:** 2024-01-29

**Authors:** Tom R. Marshall, Maria Ruesseler, Laurence T. Hunt, Jill X. O’Reilly

**Affiliations:** 1 Centre for Human Brain Health, School of Psychology, University of Birmingham, Birmingham, United Kingdom; 2 Wellcome Centre for Integrative Neuroimaging, Department of Experimental Psychology, Oxford University, Oxford, United Kingdom; 3 Wellcome Centre for Integrative Neuroimaging, Department of Psychiatry, Oxford University, Oxford, United Kingdom; 4 Wellcome Centre for Integrative Neuroimaging, Nuffield Department for Clinical Neurosciences, Oxford University, Oxford, United Kingdom; McGill University, CANADA

## Abstract

Animals actively sample their environment through orienting actions such as saccadic eye movements. Saccadic targets are selected based both on sensory evidence immediately preceding the saccade, and a “salience map” or prior built-up over multiple saccades. In the primate cortex, the selection of each individual saccade depends on competition between target-selective cells that ramp up their firing rate to saccade release. However, it is less clear how a cross-saccade prior might be implemented, either in neural firing or through an activity-silent mechanism such as modification of synaptic weights on sensory inputs. Here, we present evidence from magnetoencephalography for 2 distinct processes underlying the selection of the current saccade, and the representation of the prior, in human parietal cortex. While the classic ramping decision process for each saccade was reflected in neural firing rates (measured in the event-related field), a prior built-up over multiple saccades was implemented via modulation of the gain on sensory inputs from the preferred target, as evidenced by rapid frequency tagging. A cascade of computations over time (initial representation of the prior, followed by evidence accumulation and then an integration of prior and evidence) provides a mechanism by which a salience map may be built up across saccades in parietal cortex. It also provides insight into the apparent contradiction that inactivation of parietal cortex has been shown not to affect performance on single-trials, despite the presence of clear evidence accumulation signals in this region.

## Abstract 2

How does what you know influence where you look? This study shows that parietal brain regions both resolve competition between options (‘where should I look next?’), and—via a different mechanism—integrate information from earlier experiences (‘what have I seen before?’).

## Introduction

Far from being passive recipients of sensory information, both humans and animals actively sample the environment using their sensory organs. In rodents, active sampling processes include whisking and sniffing; in primates, the most important and best-studied process is the control of saccadic eye movements.

As an observer views a visual scene, several saccadic eye movements per second are generated in order to direct the eye’s small focal window to points of potential interest. However, each sample does not stand alone—instead, information from multiple fixations is integrated to construct a “model” of the full visual field [1,2], and this model, in turn, acts as a “prior,” influencing the selection of targets for future saccades [3]. Therefore, the process of active sampling may be viewed as an interplay between 2 concurrent processes with distinct characteristics:

Firstly, the brain must generate each individual sampling action. Since only one saccade is made at once, each individual saccade must be selected by a process of *competition* between representations of alternative possible saccadic targets [4]; the process is by necessity *winner-take-all* in that the eyes can only fixate one location at a time [5] and must operate on a fast timescale, driven by dynamics that ensure a new saccade is made every few hundred milliseconds [6].
Secondly, the brain must *integrate* the limited information gained from many individual saccades—not only to construct the visual scene but relatedly to inform the selection of targets for future saccades. Behavioural modelling suggests that the likely information value of future saccades is represented in a salience map [7,8] that can be regarded as a Bayesian prior distribution over potential saccadic targets [3,9]. Far from having winner-take-all dynamics, this salience map must capture the *relative or probabilistic distribution* over all possible saccades. It therefore must integrate information across multiple previous saccades.

The neural processes underlying the selection of individual saccades are, due to a combination of electrophysiological and modelling work, relatively well understood [10–13], but the representation of the prior is less so; the latter is the focus of the current study.

The mechanism by which candidate saccadic targets “compete” has been elucidated using evidence accumulation paradigms, designed to extend the saccade selection process over hundreds of milliseconds—most commonly random dot kinematograms (RDKs) or “moving dots” tasks [14]. Spatially selective neurons in frontal and parietal cortical eye fields (FEF and LIP) track the accumulation of evidence in favour of a saccade to their response field [10–13]. Such evidence-tracking activity is consistent with a model in which the neuronal population activity at a given moment represents the log odds that the preferred location will be the target of the next saccade, effectively ramping up to target selection or down to target rejection. The process could arise from ramping at the level of individual neurons [10,15] or from the gradual accrual of “step-changes” in individual neuronal activity [16] and can be described mathematically as a sequential probability ratio test [12]. A key element of this decision model is a winner-take-all competition between targets [17]. An influential biophysically specified form of this model developed by Wang and colleagues describes how 2 pools of neurons compete with each other to determine the choice of saccadic target [18,19]. In the present study, we use the Wang model to identify MEG signatures of the saccade selection process for each individual saccade.

In contrast, the mechanisms by which a prior or model is built up across multiple saccades is less well understood. A prior could be implemented by many possible neural mechanisms. One such mechanism is via modulation of the baseline firing rate of target-selective neurons; this has been observed in monkey LIP. However, such an “active” representation of the prior would be energetically costly over longer timescales [7,20]. A second candidate mechanism has been proposed, in which synaptic weights from neurons in the “input layer” (visual cortex) to the layer representing the prior are adjusted to favour different sensory inputs [18]. This process, which is largely activity silent in the absence of stimulation but results in a modulation of sensory input gain, would be an energy-efficient way to store priors over longer timeframes.

To probe changes in input gain relating to prior beliefs, we developed a novel approach using human neuroimaging (magnetoencephalography (MEG)), using the method of rapid frequency tagging. We reasoned that a change in input gain, such as one evoked by a change in synaptic weights [18], should result in a change in gain for even irrelevant sensory stimuli colocated with favoured saccadic targets. In rapid frequency tagging, an irrelevant, invisible perceptual manipulation (high-frequency rhythmic flicker of the targets), known to produce strong increases in oscillatory power in sensory cortices [21,22], is presented. These “tag” oscillations propagate forward through the visual system, thus indirectly probing input gains to higher visual areas such as posterior parietal cortex. Importantly, 2 competing saccadic targets can be tagged with different frequencies so that the representation for the 2 targets can be precisely separated as it propagates forward through the visual system, even if populations of cells tuned to the different targets are partially or completely spatially intermixed. This approach is therefore sensitive to prior beliefs encoded via synaptic plasticity [18] rather than neural firing rates.

Although cortical regions concerned with saccade selection exist in both frontal cortex (frontal eye field (FEF)) and parietal cortex (lateral intraparietal area (LIP)), this study focusses on the parietal cortex. The reason for this is mainly practical, as the visual frequency tag signal is attenuated as it propagates forward in cortex and is not detectable in the frontal lobe. However, we note that recent studies using cortical inactivation in rodents and monkeys suggest a key role of parietal saccade regions in construction of the prior. Rats with parietal inactivation showed a reduced influence of previous trials on interval judgements, and parietal neurons were shown to represent information about recent trials [23]. In contrast, inactivation of frontal eye fields (frontal orienting fields in the rat) affects performance on single trial target selection, but inactivation of parietal cortex does not [24,25]. Therefore, the parietal cortex is a strong candidate for the neural substrate of the prior for saccadic selection.

## Results

**Fig 1:**
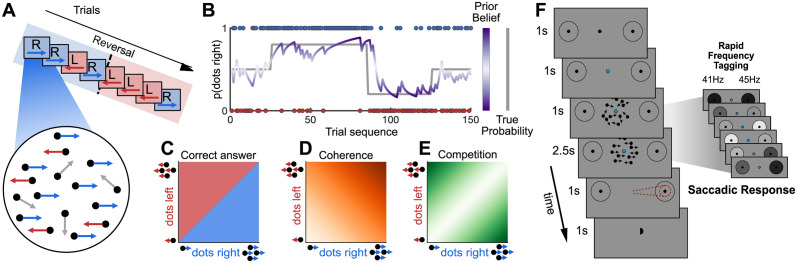
Adapted dot-motion task. (**A**) Trial sequences were presented where certain motion directions were more common, with unpredictable “reversals” (top), and stimuli within a trial varied along 2 orthogonal dimensions; number of dots moving left and right, with all other dots moving randomly (bottom). (**B**) The cross-trial prior probability p(correct direction = right) varied across the experiment, with pseudo-random, unsignalled “blocks” of trials in which the dominant direction was rightwards 20%, 50%, or 80% or the time (grey line). Bayesian learning models were used to estimate direction and strength of beliefs and observer should have about the current trial based on previous trials (purple line). (**C**) On a given trial, the correct response is given by the dominant motion direction. (**D**, **E**) Additionally, 2-d stimulus space can also be parameterized as varying along 2 dimensions: **total coherence** (middle panel), or the total percentage of coherent motion to the left or right, and **competition** (right panel), the unsigned difference between proportion of coherent dots moving left and right. (**F**) Structure of a single trial: A get-ready cue indicated dots were about to appear. All trials began with 1 second of random motion, which temporally separated stimulus onset from evidence onset, meaning we were able to distinguish visual evoked neural responses from evidence accumulation processes. This also provided a temporally extended foreperiod in which neural activity reflecting prior beliefs could be measured. Random motion was followed by a 2.5-second evidence accumulation period, in which coherent motion was present. When the dots disappeared, participants had a 1-second response interval to make a saccade in the direction of perceived dominant motion. Participants were given unambiguous feedback on the correct answer (small centrally presented hemisphere on the correct side), so they could learn the cross-trial prior independently of the quality of evidence on each trial. During the 1-second foreperiod and 2.5-second period of evidence accumulation, the 2 potential saccadic targets were “tagged” with high frequency flicker to selectively entrain neural oscillations.

The classic RDK task [26] has previously been used to show the modulatory effects of both probabilistic cueing [27,28] and choice history [29,30] on evidence accumulation. We modified this task in 2 ways (Fig 1A) to generate distinct predictions about the neural signatures of both the competitive process of selecting a single saccade and the integrative process of constructing and representing the prior.

In the classic RDK task, participants observe mixtures of *randomly* and *coherently* moving dots and accumulate evidence over hundreds of milliseconds to determine the direction of coherent motion, responding with a congruent saccade. We introduced longer-term, *cross-trial integration* favouring left or right response, to drive the computation of a prior hypothesised to occur in parietal cortex. Additionally, we introduced *within-trial competition* between left and right options so that the strength of evidence for a target could be dissociated from the direction of evidence (and, therefore, its concordance with the prior).

*Cross-trial integration (prior)*. In classic RDK tasks, the dot direction on each trial is independent. Each option (left, right) is equally likely, and so participants do not have to retain any information about the current trial after the trial ends. In our modified version, we introduced long-term correlations in the dot-motion direction. The probability that the next correct choice would be “right” was not fixed at 50% but took values of 20%, 50%, or 80% for blocks of about 25 trials (changes in prior probability were unsignalled and occurred with a uniform hazard rate of 0.04 (Fig 1B, “true probability” (solid grey line)). Since the dominant direction on previous trials could be useful for determining the direction on the current trial, participants could benefit from integrating information across trials to construct a *prior* over the dominant motion direction on the next trial (Fig 1B, “prior belief” (purple trace)). We modelled this evidence integration process using a Bayesian ideal observer model (similar to [31,32]), which captured local variations in prior probability, and learning delays. As a summary measure for the strength of the prior belief (i.e., the extent to which the prior favoured leftwards or rightwards motion), we used the expected value from the Bayesian ideal observer (see Methods, Eq 5) of p(rightward = correct), denoted *E(q*_*t*_*)*. This allowed us to test whether these prior beliefs were reflected in brain activity, either as an influence on the decision process itself or in the 1-second foreperiod prior to evidence presentation on each trial (Fig 1F).

*Within-trial competition*. In classic RDK stimuli, a single set of coherent dots move to the left or right. In our modified version, all trials included some level of evidence for *both* choice options (left and right) concurrently, and participants reported the *dominant* motion direction (Fig 1C). This means that the level of the signal-to-noise or *total coherence* (total number of left and right dots, compared to random dots, Fig 1D) was manipulated orthogonally to *competition* (ratio of left to right dots, Fig 1E). Therefore, the strength of evidence on a given trial (the precision of the likelihood function in Bayesian terms) was dissociable from its direction, which could be compatible or incompatible with the prior. This allowed us to test (behaviourally and neurally) for evidence that the prior was used and precision-weighted against current evidence strength. This manipulation also allowed us to localise in time (and space) the single-saccade decision process by testing for the hallmark of a competitive decision process, namely, that activity should depend upon the strength of evidence for the losing option as well as the evidence for the winning option [17]. In particular, we used Wang’s biophysical model of a drift diffusion-like competitive process to model decision-making in parietal and frontal cortex [33]; this model makes precise predictions about the independent effects of competition and coherence on brain activity [34]. These predictions, which were borne out in the MEG data, provide a temporal context in which signals relating to the prior may be interpreted.

Each trial began with a “get ready” cue, followed by 1 second of fully incoherent motion, followed by 2.5 seconds of coherent motion to both left and right that varied over trials (Fig 1F). The purpose of the 1-second incoherent motion period was 2-fold: Firstly, to allow signals pertaining to cross-trial integration to appear prior to evidence accumulation; secondly, to dissociate the onset of evidence accumulation from the onset of visual stimulation, which produces strong evoked activity in posterior brain regions.

### Choice behaviour is influenced by the prior in accordance with Bayesian theory

Participants (*n* = 29, final analysis *n* = 26, for information on participant exclusions, see Methods) performed 600 trials of a modified random-dot motion task with added within-trial competition and cross-trial integration, divided into 4 blocks with short breaks, while MEG was recorded. All participants also completed a practice session of 300 trials, outside the scanner, on a separate day.

**Fig 2:**
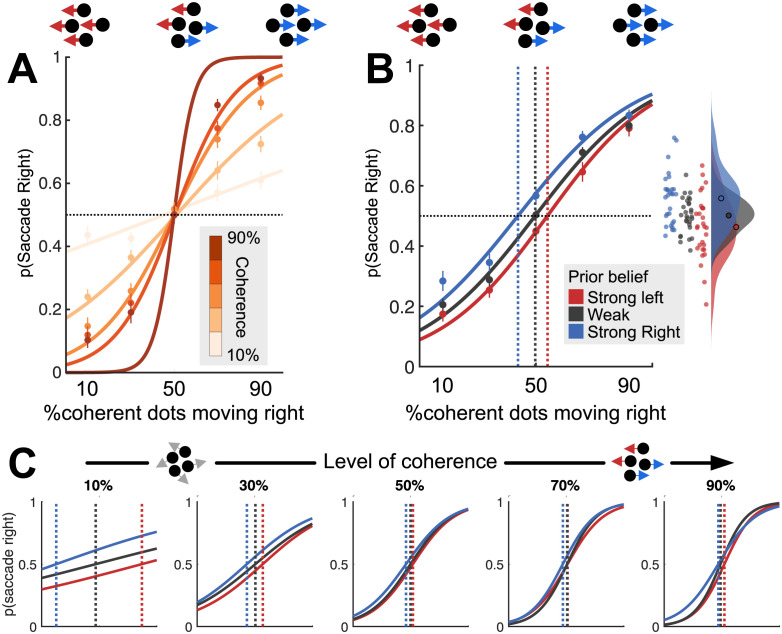
Within- and across-trial influences on behaviour. (**A**) Increasing overall coherence (darker lines) produces a parametric performance improvement (steeper logistic curve). Dots indicate mean observed data; lines indicate logistic fits. (**B**) Prior belief influences choice behaviour on current trial, leading to a shift in the point of subjective equality (dashed vertical lines). Inset: Raincloud plot showing values of p(Saccade right) for individual participants on trials where 50% of dots moved right. (**C**) Prior belief shifts point of subjective equality (dashed lines) most strongly when the least information is available on the current trial (coherence is lowest).

We first confirmed that participants’ single-trial choice behaviour was influenced by both the total coherence (signal to noise) and the competition between left- and rightward motion, by fitting logistic regressions to participants’ saccade directions (left, right) as a function of percent coherent motion and proportion of coherent dots that moved right. As expected, there was an interaction such that participants were more likely to saccade rightwards when a greater proportion of the coherent dots moved rightwards and this effect increased as the total amount of coherent motion increased (t(25) = 9.74, *p* < 2 * 10^−10^, Fig 2A).

Next, we tested whether participants learned and used the across-trial regularities in the stimulus sequence (the prior).

We first confirmed that feedback from previous trials influenced participants’ decision on the current trial using lagged logistic regression (S1 Fig), indicating that participants were indeed retaining information across at least 2 previous trials. To model participants’ prior beliefs, we used a Bayesian ideal observer model [31] (see Methods for full description) to compute, for every trial in a stimulus sequence, the *prior belief* that an ideal observer should have, based on the feedback observed on previous trials. The “ground truth” or generative probability of rightwards motion was either high (80%), low (20%), or neutral (50%); these probabilities changed unsignalled about every 25 trials (see Fig 1 and Methods). The Bayesian ideal observer model estimated the probability that the dominant motion direction on the upcoming trial would be right or left, based on the feedback from previous trials. The advantage of this approach over using “ground truth” prior probabilities is to capture local fluctuations in probabilities, and learning delays. In all analyses, the “prior belief” is defined as the expected prior probability of rightwards motion from the Bayesian model, which varies smoothly throughout the range 0 to 1. This quantifies the strength of the participant’s expectation for rightwards (versus leftwards) motion, which corresponds to the extent to which a saccade is prepared to the rightwards (versus leftwards) target.

For visualisation (Fig 2B), we divided trials using a tertile split according to whether the Bayesian prior strongly favoured rightward or leftward motion, or neither (neutral prior). On “neutral prior” trials, the point of subjective equality (PSE) closely matched the point of *objective* equality (mean 49.7% right motion). Strong priors in either direction biased the PSE by 10% to 15% (strong left prior; PSE 55% right motion, strong right prior; PSE 42.3% right motion, Fig 2B, inset).

A multiple logistic regression confirmed that participants’ choice behaviour was influenced both by the proportion of dots moving right (*t* test on regression coefficients across the group: t(25) = 14.79, *p* < 1 * 10^−14^) and by the prior based on previous trials (t(25) = 2.80, *p* = 0.0091). These 2 effects did not interact (t(25) = 0.45, *p* = 0.66). This is evidence that decision-relevant properties of the currently viewed stimulus (the proportion of coherent dots that moved right), and the beliefs participants had developed based on previously viewed stimuli, made independent contributions to their choice behaviour.

Because our bidirectional stimulus dissociated strength of evidence (total coherence) from direction of evidence (left versus right), we could test whether participants relied more upon the prior when evidence in the current trial was weak [35] (low total coherence), due to precision weighting, as predicted by Bayesian theory [35]. To visualise this effect, we repeated the Bayesian ideal observer analysis but divided the trials according to the level of total coherent motion on the current trial (Fig 2C, using the same tertile splits as above). As predicted, prior belief biased current choice strongly when there was little decision-relevant motion (at 10% coherence, PSE moved from ≈50% to 10%/90%, Fig 2C, left) but had little effect when a lot of motion was decision relevant (at 90% coherence, PSE moved from ≈50% to 48%/52%, Fig 2C, right).

The effect shown in Fig 2C was statistically confirmed using logistic regression. Percent total coherent motion had opposite effects on the influence of current evidence and prior belief on choice; when total coherent motion was high, the current evidence (proportion of coherent dots moving right) influenced behaviour more (Wilcoxon test on logistic regression coefficients for (coherence * prior) interaction across the group: Z = 4.7, *p* < 3 * 10^−6^, nonparametric test due to first-level outliers; see Methods) but prior belief influenced behaviour less (Z = −2.52, *p* = 0.012). This contrasts with the previous analysis where we found no interaction between the prior belief and the degree to which *competition* influenced choice behaviour. This confirms that participants selectively weighted the 2 sources of evidence available to them; up-weighting the impact of their prior belief—shaped by what they had seen *previously*—when little sensory evidence was *currently* available to guide their choice. This precision-weighting resembles the optimal Bayesian strategy for the task [35].

### Neural models and neuroimaging

Next, we turned to our MEG data for evidence of the neural mechanisms underlying the competitive process of selecting an individual saccade and the integrative process of forming a prior across many saccades.

### Biophysical model of the neural mean field

We simulated the neural mean field (summed activity of neuronal pools representing the left and right targets) during the saccade selection process using an adaptation of the decision model previously described by Wang and colleagues [33,34]. Because Wang’s model describes the summed activity of all neurons in the decision-making population—the “neural mean field”—it generates useful predictions about the population activity of a brain region measured by MEG. Indeed, this model has been successfully used in a value-based choice task [34] to predict the independent effects of total value (analogous to total coherence in our task) and value difference (analogous to competition in our task) on the event-related field (ERF) as measured in MEG and has previously been fit to brain activity in both parietal [33] and frontal cortex [34,36,37]. We adapted a neural mean-field version of this model to make predictions about how neural responses would vary with competition, coherence, and prior belief in our task.

Briefly, the model comprises 2 neuronal pools coding for different choice options, with strong recurrent excitation within a pool and strong inhibition between pools. The between-pool inhibition mediates a winner-take-all competition between options resulting in one pool reaching a high-firing attractor state and the other pool a quiescent attractor state. In the standard version of the model, inputs to the 2 pools are intended to be proportional to the strength of the input stimulus; in our case, number of dots moving left and number of dots moving right.

The key feature of this model is that, due to the *competition* between pools, the decision variable depends on the strength of evidence for the *unselected* option, as well the selected option [17]. We were able to directly test for the influence of the unchosen option because in our modified random dots task, evidence for the chosen and unchosen dots direction was manipulated independently, allowing us to identify signals driven by competition independently of signal to noise. The model, which uses biologically based parameters, makes specific predictions about the form and timing of neural responses, which could be tested directly in the MEG mean field.

**Fig 3:**
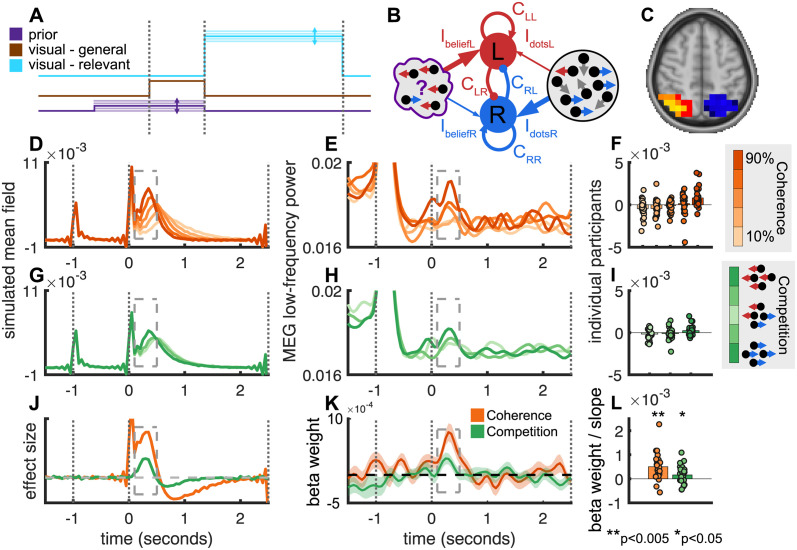
(**A**) Input to neural mean-field model. Each node received weak variable input corresponding to prior knowledge (purple), weak fixed input corresponding to motion in all directions (brown), and strong variable input corresponding to coherent motion (red). For each input, the baseline level is zero (offset for the visibility of the different traces). (**B**) Properties of neural mean-field model on a representative trial. Each node contained recurrent excitatory connections and inhibitory connections to the other node and received driving inputs corresponding to the strength of prior belief and to the properties of the dot stimulus. (**C**) Region of interest used to reconstruct activity in IPS (based on a combination of data and anatomy—see Methods). (**D**) Neural mean-field model predicts a parametric increase in activity as a function of stimulus coherence. (**E**) Low frequency in IPS activity displays parametric modulation as a function of coherence. Note: To focus on the evidence-accumulation signal following time point 0, the y-axis has been truncated, obscuring the larger response to incoherent motion onset following time point −1. A zoomed-out version of the same figure can be found in the Supporting information (S3A Fig). (**F**) Time-averaged mean field from the period indicated by the box in (**E**). Each dot is an individual participant. An expanded version of the same figure showing individual slopes, and group medians as well as means can be found in the Supporting information (S3C Fig). (**G**) Neural mean-field model predicts a parametric increase in activity as a function of stimulus competition. (**H**) Low frequency in IPS activity displays parametric modulation as a function of competition. Note: A zoomed-out version of the same figure can be found in the Supporting information (S3C Fig). (**I**) Time-averaged mean field from the period indicated by the box in (**H**). Each dot is an individual participant. An expanded version of the same figure showing individual slopes, and group medians as well as means can be found in the Supporting information (S3D Fig). (**J**) General linear model reveals effects of both coherence and competition in neural mean-field model in the poststimulus period. (**K**, **L**) Significant effects of coherence and competition are observed in IPS in the same poststimulus window. The data underlying this figure can be found in Zenodo (DOI: 10.5281/zenodo.10209832).

The neural mean-field model predicted that, firstly, increasing total *coherence* (sum of dotsL and dotsR) should produce a parametric increase in neural activity following target onset (Fig 3D) in the time window 100 to 500 ms following onset of coherent motion. Secondly, as *competition* decreases (i.e., greater absolute difference between dotsL and dotsR), there should be a parametric increase in neural activity following target onset (Fig 3G) in the same time window.

### Modelling the prior

Prior beliefs about dot direction could be built into this model as a driving input. To model this possibility (for which we ultimately failed to detect evidence in the MEG signal), we adapted the model to include *three* different input sources in different time windows (Fig 3A and 3B): The first input represented a participant’s prior belief about the upcoming stimulus (purple, Fig 3A and I_belief_, Fig 3B), the second reflected undifferentiated activity due to random dot motion during the 1-second “incoherent motion” epoch (brown, Fig 3A), and the third reflected properties of the stimulus itself—i.e., the number of dots moving left and right—during the 2.5-second “coherent motion” epoch (cyan, Fig 3A and I_dots_, Fig 3B). The timing of the driving inputs reflects electrophysiological findings that the initial response of parietal neurons [38,39] to target presentation, prior to evidence accumulation, weakly reflect the influence of prior beliefs on saccade selection [18,40]. Importantly, while the second input “general motion,” input was fixed across trials, the inputs corresponding to prior and to task-relevant motion were variable; Fig 3B illustrates a trial where the visual stimulus contains more rightward than leftward motion (right side; many right-moving dots, strong input to “right” accumulator, few left-moving dots, weak input to “left” accumulator), but the model expects leftward motion (left side; expectation of left-moving dots, strong input to “left” accumulator, weak input to “right” accumulator).

The model predicts that weakly increasing input due to prior knowledge in the prestimulus period (Figs 3A and S2A) should (a) slightly alter prestimulus activity (although the change was not significant in our simulations) and (b) bias the initial conditions of the competitive accumulation process such that activity evoked by the much stronger stimulus-driven input parametrically increased with prior strength. This biasing of the accumulation process occurred in our simulations, even though the prior input was no longer active at the time of evidence accumulation, presumably due to the weak prior input biasing the state of the network before the stronger inputs began.

### Event-related activity reflects the competitive process of selecting the current saccade

We next tested whether the pattern of results predicted by the neural mean-field model were observed in MEG data from the parietal cortex, which is known to track evidence accumulation. We transformed participants’ MEG data to source space using linearly constrained minimum variance (LCMV) beamforming [41] and extracted time series from regions of interest (ROIs) in parietal cortex (Fig 3C, see Methods for ROI definitions). We extracted the time-varying power in the low frequency range (2.8 to 8.4 Hz) as a proxy for the event-related field or ERF, the magnetic field arising from local field potentials in cortex [42]; this was necessary (rather than calculating the ERF directly) because the beamforming (source localisation) procedure introduces arbitrary sign-flips in the ERF, but no such ambiguity in the time-frequency domain. For further details see, Methods/Parietal cortex and frontal eye field low-frequency ROI analysis. This analysis revealed 2 transient responses (Fig 3E, 3F, 3H and 3I); the first shortly after onset of incoherent motion, the second 100 to 500 ms after onset of coherent (i.e., choice-relevant) motion (Fig 3E and 3H, dashed boxes).

Concordant with ramping activity observed in electrophysiological studies [10,12], there was a clear parametric modulation of the low-frequency MEG signal 100 to 500 ms after the onset of coherent motion as a function both of total coherence (Fig 3E and 3F) and stimulus competition (Fig 3H and 3I). As predicted, stronger evoked activity was observed with higher levels of total coherence; this is consistent with the observation that ramping evidence-accumulation signals in single-unit studies rise faster for higher coherence levels in standard dot motion tasks [12], whether those signals arise from ramping in individual neurons or from step-changes in neurons leading to a population-level ramp [16]. Stronger evoked activity was observed for *lower* levels of stimulus competition, indicating that the presence and strength of evidence conflicting with a decision affects the decision process—a hallmark of a competitive system. Statistically, a linear multiple regression with parameters coherence, competition, and prior strength confirmed that the amplitude of the evoked response varied as a function of both coherence and competition; specifically, and as predicted by the neural mean-field model (Fig 3J) activity increased as a function of stimulus coherence (t(25) = 4.67, *p* = 9 * 10^−5^), and decreased as a function of competition (t(25) = 2.18, *p* = 0.039, Fig 3K and 3L). Both effects were tested in a time window 100 to 500 ms after the onset of coherent motion (dashed boxes), defined based on the predictions of the biophysical model.

Since ramping activity is also observed in other brain regions such as FEF, we repeated the analysis in this region (S4 Fig). The pattern of results was qualitatively similar. To test whether there was any difference in timing of effects between FEF and parietal cortex, we used permutation testing (permuting the waveforms between ROIs within participants) and found no significant difference in timing between regions (coherence; *p* = 0.12, competition; *p* = 0.92).

The qualitative correspondence of the MEG activity to a neural mean-field model of a competitive decision process suggests that parietal cortex could be engaged in resolving saccadic choices via competition by mutual inhibition, compatible with previous observations of evidence accumulation signals. However, it is worth noting that evidence from inactivation studies [24,43] suggests that the activity in parietal cortex is not causally necessary for saccade selection—whereas activity in FEF is. Although more recent studies do suggest LIP may play a causal role in evidence accumulation [44], the behavioural effects of targeted inactivation are somewhat transient (despite persistent neural inactivation), which—at a minimum—suggests the existence of parallel or compensatory mechanisms. The signal observed in parietal cortex may, we hypothesised, play a parallel role in integrating this information over a longer timeframe into a cross-saccade prior.

### Effects of the prior on the mean field

Contrary to our model predictions, prior belief did not significantly modulate the low-frequency MEG signal in parietal cortex (t(25) = 0.80, *p* = 0.43, S2 Fig). However, the absence of an effect of prior should be interpreted with caution, as for any null result; perhaps the effect was too subtle to be detected in the present paradigm. Furthermore, a relevant theoretical model [18] predicts an interaction between signed evidence on the current trial and signed prior belief based on previous trials. Accordingly, we tested for this interaction; however, no significant effects were observed in the low-frequency MEG signal in parietal cortex (left hemisphere; t(25) = -1.51, *p* = 0.14, right hemisphere; t(25) = 0.087, *p* = 0.93).

### A prior integrating across multiple saccades is implemented in parietal cortex via gain modulation

Although participants’ behaviour was clearly influenced by information on previous trials (S1 Fig) consistent with computing a prior (Fig 2B), we were unable to detect activity corresponding to the prior in low-frequency MEG signal (approximating the ERF, mainly driven by synchronised postsynaptic potentials).

We then turned to the possibility that the prior is implemented via modulation of sensory gain, as first proposed by Soltani and Wang [18]. Sensory gain modulation is defined as a process that changes the slope of the relationship between a driving input (in this case, a visual stimulus flickering at a high frequency) and neural activity (in this case, a signal at the corresponding frequency in parietal cortex).

To probe for possible gain changes between visual inputs and the parietal cortex, we exploited the method of rapid frequency tagging [45]. During the time that moving dot stimuli were present on the screen, the 2 saccade targets were rhythmically flickering at 2 different high frequencies (41 and 45 Hz, Fig 1F) that were both task irrelevant and indistinguishable to observers. Flicker at such high frequencies is typically not perceived (they are, for example, close to the refresh rate of a standard 60-Hz computer monitor—note that, here, we used a 1.44-kHz projector to present stimuli). Indeed, no participant reported awareness that the stimuli were flickering. Flickering stimuli have previously been shown to produce detectable rhythmic activity in M/EEG signals [46], including at higher frequencies above 40 Hz [21,22], presumably by producing synchronised neural activity in visual cortex that propagates through the visual streams [47,48].

**Fig 4:**
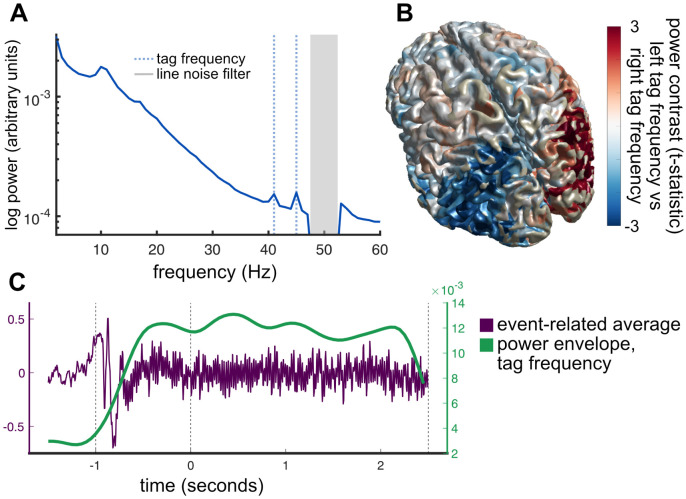
(**A**) Average Fourier spectrum of left and right parietal cortex ROIs displays clear peaks at the 41-Hz and 45-Hz tagging frequencies (dashed blue vertical lines). Note that grey box indicates line noise filter. (**B**) Unthresholded statistical map of differences between “power at left tag frequency” and “power at right tag frequency.” Only parietal and lateral occipital regions showed lateralised differences in tag frequency power, presumably due to weaker SNR in frontal sources. (**C**) Illustration of frequency tagging in a representative participant. Onset of moving dots and visual flicker at saccade targets produces detectable high-frequency activity in the timelocked-average MEG signal (purple trace). Sliding-window Fourier analysis tracks the power envelope at this frequency (green trace).

Parietal neurons are known to increase their sensory input gain when an attentional or saccadic target is present in their receptive field [11,49–51]. We reasoned that such a change in gain would result in a retinotopically localised increase in the propagation of information from visual to parietal cortex, including task-irrelevant information such as the frequency tags. When one or other target is expected to be the saccadic target (for example, due to a prior belief), we would expect the tag frequency for that target to propagate more effectively into parietal cortex. We would expect the effect to be seen mainly in the contralateral hemisphere due to the lateralized representation of the visual field in occipital cortex, but, importantly, the detection of a lateralized prior does not depend upon lateralisation of the MEG signal (as spatial specificity in MEG is relatively weak) but on detection of a specific frequency (which can be done with excellent precision in MEG—see Fig 4A): Because the 2 saccadic targets were tagged with different frequencies, any increase in gain for the retinotopic region containing one of the saccadic targets would be reflected in a *frequency-specific* change in the signal in parietal cortex.

#### Validation of frequency tagging

In the MEG data, we recovered clear spectral peaks at the 41-Hz and 45-Hz tag frequencies (Fig 4A) that were present while the tag stimulus was active. There was a clear lateralized effect such that the tag frequency presented at the right target was strongest in the left hemisphere of the brain, and vice versa.

The frequency tagging effect was strongest in parietal and occipital regions, propagating throughout posterior parietal cortex (Fig 4B), detectable from about 500 ms after flicker onset (see Methods) and persisted during the entire stimulus period (Fig 4C). It should be noted that the frequency tagging signal could not be detected in frontal cortex including FEF. This was expected given that the effect is an entrained visual oscillation—the signal propagates forward from occipital cortex but only through a few synapses. Assuming some degree of postsynaptic “jitter,” more synapses between visual cortex and target region would necessarily lead to more jitter, and reduced signal, to the point where no tagging activity could be observed.

We defined “frequency tag activity” for each parietal ROI (left, right) as the time-resolved power at the specific flicker frequency of the contralateral flickering saccade target, corrected for the main effects of flicker frequency and hemisphere. Notably, our parietal ROI overlapped substantially with 2 ROIs thought to be homologous to eye movement regions LIP and 7a in the macaque [52]; however the spatial resolution of MEG is not sufficient to say with certainty which intraparietal subregions were the source. For full description of ROI definition, see Methods.

For comparison, we also examined frequency tag activity in occipital cortex. If the prior is indeed implemented as a gain modulation between the input layer (visual cortex) and the parietal cortex, we would expect to see task-dependent modulation of the tag signal in parietal cortex, but not occipital cortex.

#### The cross-saccade prior is represented prior to evidence accumulation

If the parietal cortex represents a prior expectation based on the integration of previous saccades, this should be in evidence in the foreperiod, when only incoherent motion was present. We defined a time window from 500 ms after flicker onset (the point at which tag activity was first evident in parietal cortex, Fig 4) until the onset of coherent motion. We divided trials with a tertile-split into those on which the prior strongly favoured the contralateral target, strongly favoured the ipsilateral target, or was close to neutral.

**Fig 5:**
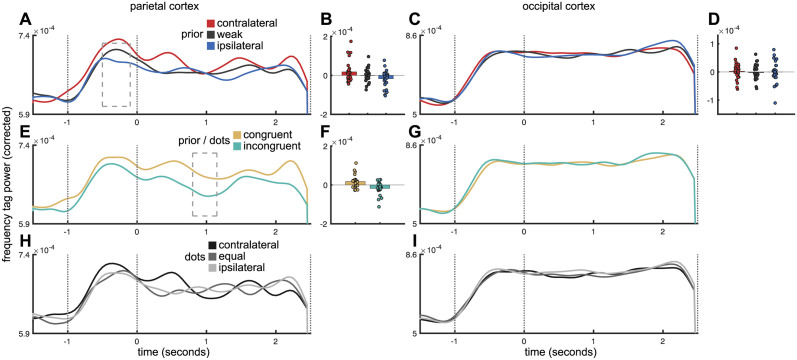
(**A**) Time course of frequency tagging activity in parietal cortex as a function of prior belief (contralateral, weak, or ipsilateral to region). (**B**) Direction of prior belief modulates parietal activity before onset of coherent motion. Colour coding as for (**A**). Dots represent individual participants. (**C**, **D**) As (**A**, **B**), but for occipital cortex. (**E**) Time course of frequency tagging activity in parietal cortex as a function of congruence of prior belief and dominant direction of moving dots (congruent, incongruent). Dashed box indicates time window where cluster values were maximal. (**F**) Individual participant data points averaged across time window denoted by cluster test. (**G**) As (**E**), but for occipital cortex. Note, no individual data points are plotted as nonparametric cluster permutation revealed no clusters. (**H**) Time course of frequency tagging activity in parietal cortex as a function of dominant dot-motion direction. No significant dot-motion–related activity modulation was observed. (**I**) As (**H**), but for occipital cortex. No significant modulation was observed. The data underlying this figure can be found in Zenodo (DOI: 10.5281/zenodo.10209832).

Concordant with our hypothesis, we found that during the foreperiod, frequency tagging activity reflected the direction of the prior—activity was strongest on trials when the prior favoured the contralateral target (linear contrast comparing tertiles strong-contra, weak, and strong-ipsi: t(25) = 2.27, *p* = 0.028, Fig 5A and 5B). No such effect was observed in occipital cortex (t(25) = -0.223, *p* = 0.824, Fig 5C and 5D).

To test for a parametric effect of prior belief on frequency tag strength, we conducted a linear regression of tag strength on prior belief, defined as the expected value (from the Bayesian model) of the prior probability of contralateral motion on the upcoming trial, p(contra)). This failed to reach significance (group *t* test on regression coefficients, (t(25) = 1.41, *p* = 0.085). The difference between parametric and categorical analyses is small and should perhaps not be overinterpreted; it implies that the relationship between frequency tagging and prior strength may be somewhat nonlinear (such that the difference between leftward and rightward priors is greater than similar sized differences within each category), but to determine the shape of this relationship is beyond the sensitivity of the current dataset.

#### Gain modulation in parietal cortex is sensitive to dot motion, in the context of the prior

While the frequency tag data provided evidence of gain modulation relating to prior beliefs, it did not track evidence accumulation (coherence or competition on a single trial basis) in the same way as the ERF (S5 Fig). We note that, as the build-up of activity during evidence accumulation reflects recurrent excitation, the dynamics of which are determined by the network properties of parietal cortex [53], we would not expect a high-frequency oscillation to “build up” in the same way the mean field does (for example, there may be interference between incoming and recurrent waveforms). The lack of an evidence-related change in the frequency tag signal is compatible with a model in which the prior is implemented via sensory gain modulation, which could have several possible mechanisms [54] including activity-silent synaptic plasticity [18], or by tonic changes in baseline firing rate, for example, in a bump attractor [55] since tonic activity would likely not be detected in the band-passed MEG signal.

Although our main test for a representation of the prior was whether a gain modulation signal relating to prior beliefs was observed in the foreperiod of the task (during which the only lateralized effect is prior belief), we also asked, as an exploratory question, whether at any point after the onset of coherent motion, this prior-related signal was modulated by the motion direction on the current trial. To test this, we coded trials as “congruent”—i.e., the target favoured by the prior was also favoured by within-trial evidence—or “incongruent.” Indeed, we found that stronger activity at the contralateral tagging frequency was observed on congruent than incongruent trials (Fig 5E and 5F; linear regression followed by cluster-based permutation test on regression coefficients, t_maxsum_ = 21.0023, cluster *p* = 0.0238, see Methods; no such effect was seen in occipital cortex (no cluster *p* < 0.05, Fig 5G)). Interestingly, the parietal effect was observed to be strongest in a later time window (800 to 1,150 ms after stimulus onset) than the decision-related activity. This suggests that this signal reflects a late process of integrating the evidence with the prior, after the competition between leftward and rightward dots has been resolved.

To further probe the nature of this congruency signal, in response to feedback on the manuscript, we looked at 2 more subtle measures of congruence—the precision of the belief distribution encoding the combination of the prior and the dot motion evidence (which could be considered to capture belief confidence), which we termed the *within-trial posterior*, and the KL divergence between the *within-trial posterior* and the prior, which could be taken as a metric of the degree of belief updating based on the dot-motion evidence. The within-trial posterior was obtained from an elaborated version of the Bayesian model, described at *model 2* in the Methods section. Neither of these variables predicted the frequency tag signal, in a general linear model in which the dependent variable was the mean frequency tag signal in the significant time window for the congruency effect (800 to 1,150 ms), and the prior (coded in tertiles as above), absolute and relative coherence, and congruency between prior and dot motion (as coded above) were included as control variables (t = 1.35, *p* = 0.19 for the precision of the within-trial posterior; t(25) = −0.81, *p* = 0.43 for the KL divergence).

It is worth noting that, while we might hypothesise that activity in parietal cortex would increase with the KL divergence, in accordance with the degree of “work done” to integrate prior and dot-motion evidence [31], it is much less clear how one should expect a metric of sensory gain, such as the frequency tag signal, to vary with internal computations; a similar argument can be made for the precision of the within-trial posterior. We therefore remain agnostic about the precise computational interpretation of the congruence signal, but note that as it depends upon the interaction of prior and dot-motion evidence, it is likely integrative in nature.

#### No effect of saccade direction on frequency tag signal

At no point did we observe a main effect of the ultimate saccade direction on the frequency tagging signal in parietal or occipital cortex (no clusters *p* < 0.05, Fig 5H and 5I); this is perhaps unsurprising as the frequency tagging stimulus ended at the end of the coherent motion period, several hundred milliseconds before the saccade was made.

#### No effect of task variables on frequency tagging signal in occipital cortex

To determine whether our findings were specific to parietal cortex, we repeated the analysis of prior belief, prior/stimulus congruence, and dot direction (Fig 5A, 5B, 5E, 5F and 5H) on the frequency tagging signals from the occipital cortex (Fig 5C, 5D, 5G and 5I). No effects of any task variables were observed; the effect of the prior in occipital cortex was near to zero (t(25) = −0.223, *p* = 0.824) in contrast to the significant effect of prior in parietal cortex (t(25) = 2.27, *p* = 0.028). However, we should note that in a direct comparison, the difference in the effect of the prior between parietal and occipital cortices did not reach significance (t(25) = −1.30, *p* = 0.10, one tailed). Thus, we can conclude that the prior is implemented as a sensory gain effect that affects the extent to which visual information (the 2 targets’ tag frequencies) propagate to parietal cortex but cannot say definitively where in the visual processing pathway this effect is implemented; we note that the anatomical separation of ROIs in MEG is imperfect.

Additionally, the absence of task modulation gives us confidence that we are recording separate signals that behave differently. Even though we used beamforming to separate contributions from distinct neural sources, it is possible, due to field spread, that—rather than representing independent signals from parietal and occipital cortices, respectively—the frequency tagging channels, in fact, represent 2 different measurements of the same underlying neural source. This control analysis makes that possibility unlikely.

#### Lateralisation of effects

It is notable that while the effects of prior belief observed in parietal cortex from frequency tagging (Fig 5) were lateralised, we were unable to detect lateralized effects in our analysis of the low-frequency, event-related responses (Figs 3 and S2). In that analysis, activity in parietal cortex *as a whole* resembled the neural mean-field model *as a whole* (for null analyses of lateralised effects of motion on the current trial and prior belief based on previous trials, in the low-frequency event-related MEG signal, see S6 and S7 Figs, respectively). Frequency tagging capitalises on the exquisite spectral specificity of the MEG signal—due to their differing peak frequencies, signals originating from the left and right target could be very clearly distinguished and contrasted (see the spectral peaks in Fig 4A).

### Time course of neural effects suggests a sequential interplay between the prior and evidence accumulation processes

**Fig 6:**
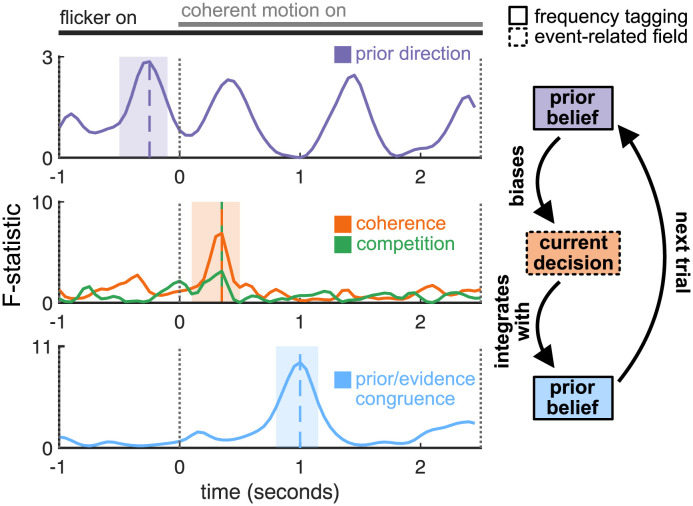
Summary of neural effects. Temporal cascade of computations. Time courses of significant statistical effects; repeated measures ANOVAs comparing contralateral, weak, and ipsilateral prior belief (top row), all levels of stimulus coherence (middle row, orange) and stimulus competition (middle row, green) and comparing “evidence (dots) congruent with belief” trials with “evidence (dots) incongruent with belief” trials (bottom row). Bottom panel shows the time point-wise repeated measures ANOVA comparing dot-prior-congruent with dot-prior-incongruent trials (even though this is a paired comparison, an F- rather than t-statistic is shown to facilitate visual comparison with the upper and middle panels. Shaded boxes indicate preselected analysis windows (top and middle rows; see Figs 3E, 3F, 3H, 3I, 4C and 4D) and window delineated by cluster permutation test (bottom row; see Fig 4E and 4F). Coloured vertical dashed lines indicate maxima of the respective F-statistics. The data underlying this figure can be found in Zenodo (DOI: 10.5281/zenodo.10209832).

An advantage of MEG over other human neuroimaging methods is its high temporal resolution. This allowed us to examine the sequence of effects concerning the selection of individual saccades and their integration into a prior (Fig 6).

To directly compare the sequence of events, we ran a series of ANOVAs plotting the effect sizes for each of the previously described factors affecting parietal activity, as a function of time. A clear sequence of events emerged: Initially, the prior is implemented via gain modulation (frequency tag effect), then the evolution of event-related activity reflects the evidence accumulation process as captured in a competitive mean field model (ERF), and, finally, the interaction of evidence and prior is again reflected in gain modulation (frequency tag effect). Before the onset of coherent motion (interval [−1 0]), differences in activity across trials must relate to the prior. In this “foreperiod” time window, we indeed observed a lateralized effect in parietal cortex as a function of the participants’ prior belief. Then, shortly after stimulus onset, we observed parametric effects of stimulus coherence and stimulus competition predicted by the neural mean-field model and likely the signature of the choice process itself. Finally, having resolved competition between choice options, we observed a later effect that related to congruence between the participants’ initial prior (i.e., a prediction about the upcoming stimulus direction) and their eventual decision, namely, a strong suppression of activity when the stimulus is incongruent with the prior belief.

The above is consistent with a model in which event-related neural activity field reflects the evidence accumulation process, but a prior integrating over multiple previous saccades and their outcomes is also implemented by the modulation on input gains in parietal cortex [18]. This prior is represented even outside the time period in which evidence for different saccades is being weighed. The representation of the prior that is present before each saccade (in the foreperiod in our experimental task) serves to bias the saccade selection process, influencing choice behaviour (Fig 2B and 2C). During evidence accumulation, competitive dynamics lead to the selection of one or other saccadic target. Once the saccade selection process is resolved (but before the saccade is physically executed), a congruency signal is observed in parietal cortex, which could reflect belief updating or evaluation of decision confidence; our paradigm is not designed to distinguish these possibilities.

## Discussion

Optimal exploration of the visual environment through eye movements requires the brain to perform 2 distinct computations: selecting among *currently* competing saccadic targets, and integrating information across saccades to construct a Bayesian prior in order to plan *future* saccades.

Using whole-brain imaging at high temporal resolution with MEG, combined with computational modelling, we demonstrated a temporal cascade of computations relating to both these processes in parietal cortex. Notably, our observations are consistent with distinct mechanisms for the processes. Competition between left- and right-targets is resolved by mutual inhibition between 2 pools of neurons driven by left- and rightwards evidence. This process produces characteristic signals relating to the strength of evidence both for the chosen and unchosen option, which can be detected in both FEF and parietal cortex. In addition, prior beliefs are represented through gain modulation in parietal cortex, in advance of evidence presentation; after the competitive process is resolved, this signal reflects the congruence between current evidence and prior beliefs.

Neurally, representing each type of task-relevant information—currently available and previously learned—requires computations that operate on different intrinsic timescales and display different neural dynamics. Resolution of competition between 2 currently available choice options requires a fast, winner-take-all neural system that rapidly converges to one of 2 stable attractor states. In contrast, representation of previously learned knowledge must maintain a broadly similar state over a long timescale and incrementally change as new data points are incorporated. It is therefore perhaps unsurprising that the 2 computations are implemented by different neural mechanisms.

Our results provide evidence for a specific mechanism by which parietal cortex has been proposed to represent prior beliefs over longer timescales [18], namely, selective modulation of the sensory gain between visual and parietal cortices. Notably, and contradicting our expectations, we did not detect effects of the prior on the mean field, although this does not rule out the possibility that such an effect occurred. Furthermore, we cannot rule out that active changes such as in baseline firing rate also occur, and, indeed, these have previously been observed [38]; as bandpass filtering is a necessary step in the MEG processing pipeline, such tonic effects would be attenuated in our signal. However, theory suggests that, when representations are sustained over long inactive periods or delays may be more efficiently represented by non-spiking mechanisms provide particularly energy-efficient ways to implement this [20]. Physiologically, gain modulations can arise from a wide range of mechanisms pertaining to synaptic dynamics, neuromodulatory factors, and the timing and position of inputs [54]. One possible mechanism is short-term synaptic plasticity on the timescale of seconds. This is reminiscent of the proposal that short-term synaptic plasticity may act as an activity-silent store for working memories [18]; the activity pattern associated with working memory is later reactivated as a bump attractor [55,56]. Indeed, a prior over saccadic targets could be viewed, in terms of timescale, as a form of noncognitive working memory.

We note that, while frequency tagging allows us a noninvasive metric of gain modulation in parietal cortex, it would be less sensitive to similar effects in frontal brain regions such as frontal eye fields; therefore, we cannot comment on whether similar mechanisms for representing the prior could be present in other saccade-planning regions of cortex.

We found evidence for both within- and across-trial evidence accumulation in parietal cortex. A wide body of literature has implicated the EEG centro-parietal positivity (CPP) in evidence accumulation [57,58] that has been hypothesised to encode an accumulate-to-bound process typically modelled with a drift diffusion model [59]. However, both the CPP and drift diffusion tend to be studied in contexts where the stimulus space in *one-dimensional*. Importantly, in our dot stimuli, some evidence for left and right motion was always concurrently present, i.e., the stimulus space was two-dimensional. For this reason, we instead derived predictions from a neural circuit model specifically designed to implement the desired two-dimensional neural dynamics [60], the predicted parametric modulations of population activity as a function of both stimulus coherence and stimulus competition, which we observed with MEG.

The method of rapid frequency tagging, while limited to regions of cortex within a few synapses of V1, can be used as a tool to probe activity silent representations in human cortex, which cannot be accessed by studying standard stimulus-evoked fields [61] and could be considered a temporally extended form of “pinging” [62]. This may be particularly germane to the study of representations constructed and maintained over slow timescales, particularly those spanning multiple task trials or events. An additional strength of the method is that, by exploiting the spectral precision of the MEG signal, the representation of stimuli “tagged” with different frequencies can be disentangled, even when neurons tuned to those stimuli are intermixed in cortical tissue.

## Methods

### Ethics statement

Ethical approval for the study was granted by the local ethics board (Medical Sciences Interdivisional Research Ethics Committee, R53476/RE004), and all participants provided written informed consent.

### Behavioural task

Participants (*N* = 34) performed a variant of the classic dot-motion task. One second after a preparatory cue, a field of 100 dots covering 4.2 degrees of visual angle (on-screen width 8.8 cm, screen distance 120 cm) was presented at fixation for 3.5 seconds. For the first second, all dots moved randomly (uniform distribution over motion directions). After 1 second, some dots changed direction and moved either to the right or to the left. There were 5 levels of overall coherence (sum of left-moving and right-moving dots): 10%, 30%, 50%, 70%, and 90%, and 3 levels of competition: Coherently moving dots moved either 90% or 70% in the direction of dominant motion, or 50% in each direction (i.e., no correct answer). Two saccade targets subtending 4 degrees of visual angle were concurrently presented at 6.8 degrees eccentricity, 0.8 degrees below the horizontal midline. Participants were instructed to maintain fixation until the dots disappeared, then to make a saccade to the saccade target on the side of dominant motion as quickly as possible. After 1 second, feedback (a semicircle at fixation) was shown indicating the correct side.

Across trials, the correct side was drawn from a generative distribution with p(right) either 20%, 50%, or 80%. The generative distribution changed randomly across a block with a fixed probability of 4%, i.e., a switch approximately every 25 trials. On trials with no correct answer (i.e., where equal numbers of dots moved left and right), feedback was drawn from the same generative distribution as the dots; however, a programming error led to the feedback in this condition being the reverse of what was intended. This error affected approximately 8% of all trials.

All participants were trained on the task in a separate session outside the MEG for 300 trials (approximately 40 minutes). Five participants were excluded due to low accuracy in the practice session, and 29 participants completed the MEG session. During MEG acquisition, data were recorded in 4 runs of max 20 minutes, each consisting of 150 trials. Total task time was approximately 80 minutes.

### Power calculation

Sample size was determined by simulation. Based on the z-scores and sample size from the MEG analysis in [34], we calculated the effect size d as the z-score divided by the square root of the sample size. We then simulated a population of 10,000 virtual participants with this effect size, randomly sampled subsets of 10 to 60 participants from this population, calculated the subsample z-score, and computed statistical power for each subsample size as the proportion of z-scores for that sample size that exceeded 1.65 (equivalent to *p* < 0.05 in a one-tailed test). Simulation results indicated statistical power of 80.5% at our final sample size of *N* = 26.

### Neural models

#### Neural mean-field model

To model decision dynamics in FEF and parietal cortex, we implemented a mean-field reduction of a spiking network model [19]. Full details of the biophysical parameters of the model are given in [34]. Briefly, the model consists of 2 connected neuronal pools each coding for one motion option (left, right), with within-pool excitatory connections and across-pool inhibition. Each unit receives noisy background inputs simulating endogenous cortical noise, plus 3 task-related inputs: Firstly, a weak (0 to 1.6 Hz) input to one pool in the prestimulus period, simulating an initial bias in the decision process due to parietal input. Secondly, a weak (5 Hz) input to both pools during the incoherent motion period, capturing the presence of low levels of motion in both decision-relevant directions. Thirdly, a strong input (ranging from 10.1 Hz to 18.1 Hz) to each pool in the coherent motion period proportional to the number of dots in each motion direction.

We focused on the synaptic inputs (I_1_, I_2_) to facilitate comparability with the MEG data, since MEG is known to be primarily sensitive to postsynaptic potentials [42]. For optimal comparison with the MEG data, which were bandpass-filtered and transformed to the frequency domain, removing the DC component, we used the temporal derivative of the signal from the mean-field model.

#### Bayesian learning model

Because the modified dots task had temporal structure (dominant motion direction on trial *j* could be predicted, but not perfectly, from trials *1… t-1*), performance could be facilitated by tracking the true generative distribution of dominant motion directions, including when it changed. To model participants’ learning strategies, we used a Bayesian ideal observer model, a “virtual participant” that was fed the sequences of feedback given to the human participants and constructed, via Bayesian inference, a belief about the generative distribution on the upcoming trial.

Two Bayesian models were constructed. Model 1 was used throughout all analyses to give the prior belief about dot motion on each trial, based on veridical feedback from previous trials. Model 2 is an elaboration of model 1, which also calculates the full posterior belief distribution on each trial *before feedback is given*, i.e., integrating prior belief and the observed dot motion on that trial. Model 2 is used in the analyses of the effect of prior/dot motion congruence on frequency tag data.

### Model 1 details

On each trial, the prior probability that the dominant motion direction would be “right” followed a Bernoulli distribution with parameter *q*_*t*_, i.e., the prior probability that the correct answer would be “right” on trial *t* was *q*_*t*_.

The prior distribution over *q*_*t*_ was initiated as a uniform on the range (0,1) on the first trial and, thereafter, obtained from the posterior over *q*_*t*−1_, based on the outcomes *x*_1:*t*−1_, combined with a uniform “leak”; the posterior and “leak” distributions were weighted by a factor H representing the true hazard rate:

$$p\left(Q_{t}=q|X_{1:t-1}\right)=\left(1-H\right)∙p\left(Q_{t-1}=q|X_{1:t-1}\right)+H∙U(0,1)$$ (1)

where

H=pqt≠qt-1=125 (2)

i.e., it was assumed that participants know approximately the true value of *H* following extensive pretraining.

The posterior *p*(*Q*_*t*_ = *q*|*x*_1:*t*_) was obtained iteratively from the prior *p*(*Q*_*t*_ = *q*|*x*_1:*t*−1_) and the likelihood:

$$p\left(Q_{t}=q|X_{t}\right)=p\left(X_{t}\right|x\sim B(q))$$ (3)

using Bayes’ theorem:

$$p\left(Q_{t}=q|X_{1:t-1}\right)\propto \;p\left(X_{t}\right|x\sim B(q))∙\;p\left(Q_{t}=q|X_{1:t-1}\right)$$ (4)

where the posterior over *q*_*t*_ was normalised to integrate to 1.

Where a scalar value for the “prior” is used in data analysis, this is the expected value of *q*_*t*_ based on the prior distribution

$$E\left(q_{t}\right)=\int p\left(Q_{t}=q|X_{1:t-1}\right)∙q\;dq$$ (5)

### Model 2 details

The prior over motion direction on each trial, *p*(*Q*_*t*_ = *q*|*X*_1:*t*−1_), was obtained as in Model 1. On each trial, the likelihood function over dot motion, *based on the observed motion* (as opposed to veridical feedback as in model 1), was calculated based on the closed form expression for the posterior of the beta-binomial model:

$$L\left(q\right)=Β(k+1,\;\left(n-k\right)+1)$$ (6)

i.e., the likelihood function over q, $L\left(q\right)$, is a beta distribution with parameters (k+1), (n−k)+1, where n is the number of observations and k is the number of hits.

In the current application, the number of observations is proportional to the proportion of coherently moving dots *n*_*R*_+*n*_*L*_, and the number of hits *k* is be proportional to the proportion of dots moving rightwards *n*_*R*_:

$$p\left(Q_{t}=q|n_{L},\;n_{R}\right)\;\sim \;Β(W(n_{L}+\;n_{R})+1,\;W(n_{L})+1)$$ (7)

where W is a free parameter for each participant, capturing the effective number of observations on which the estimate of dot-motion was based; this parameter reflects the possibility that only a proportion of coherently moving dots contribute to the dot motion decision, and by varying its weight allows the relative weighting of the observed motion versus the prior.

The posterior belief distribution (based on the prior and observed dot motion, but before the veridical feedback *X*_*t*_ is observed) is then obtained using Bayes’ theorem:

$$p\left(Q_{t}=q|X_{1:t-1},n_{L},\;n_{R}\;\right)\propto \;p\left(Q_{t}=q\right|n_{L},\;n_{R})∙\;p\left(Q_{t}=q|X_{1:t-1}\right)$$ (8)

To fit the free parameter *W*, we modelled response selection as a softmax function in which the variable determining choice was the probability (under the within-trial posterior) that rightward motion was more likely than leftward motion:

$$p\left(R\right)=\int_{0.5}^{1}p\left(Q_{t}=q|X_{1:t-1},n_{L},\;n_{R}\;\right)∙dq$$

psaccaderight=expp(R)θexpp(R)θ+exp1-p(R)θ (9)

where *θ* is the softmax inverse temperature, a second free parameter.

*W* was fitted jointly with the softmax inverse temperature *θ* using a grid-search approach to maximise model log-likelihood. The results of the fitting process are show in S8 Fig.

The precision of this posterior was then calculated as the inverse variance, and the KL divergence as

$$\sum_{q}p\left(Q_{t}=q|X_{1:t-1},n_{L},\;n_{R}\;\right)∙ln\left(\frac{p\left(Q_{t}=q|X_{1:t-1},n_{L},\;n_{R}\;\right)}{p\left(Q_{t}=q|X_{1:t-1}\right)}\right)$$ (10)

### Analysis of saccade data/behavioural data

Custom matlab code extracted saccade direction (left, right, or no saccade) based on the eyetracker data. Due to a technical error, one participant’s eyetracker data were overwritten and saccade information was reconstructed from the horizontal EOG.

To analyse saccade data, we fit logistic regression models. Firstly, we asked whether the probability of saccading to the right on trial *t* depended on the proportion of coherent dots moving right on trial *t* (%R = 10%, 30%, 50%, 70%, or 90%), the total coherence on trial *t* (coh = 10%, 30%, 50%, 70%, or 90%), and the interaction of these (%R-mean(%R) × coh) (Fig 1A).

Secondly, we asked whether the probability of saccading to the right on trial *t* depended on the proportion of dots moving right on trial *t* (%R = 10%, 30%, 50%, 70%, or 90%), the participants’ prior belief about the dots on trial *t* formed from observing feedback on trials *1*,*2… t-1 E*(*q*_*t*_) as defined in Eq 5 above), and the interaction of these (Fig 1B).

Thirdly, we asked whether the observed effects of proportion dots moving right and prior belief on saccade direction were altered as a function of the level of stimulus coherence. To test this, we fit a first-level logistic regression with evidence (%R = 10%, 30%, 50%, 70%, or 90%), and prior belief *E*(*q*_*t*_) at each level of total coherence (coh = 10%, 30%, 50%, 70%, or 90%). We then computed linear contrasts over the effects for evidence and prior belief across the 5 levels of total coherence. Due to the presence of outliers at the first level (generalised extreme studentised deviate many-outlier procedure [63]), we used nonparametric Wilcoxon signed rank tests at the second level to test against the null hypothesis of zero median.

### MRI acquisition

To enable localisation of cortical source generators of the MEG signal, a high-resolution structural MRI was acquired for each participant using a Siemens 3T PRISMA MRI scanner with voxel resolution of 1 × 1 × 1 mm^3^ on a 232 × 256 × 192 grid. The anatomical MRI scan included the face and nose to improve coregistration with the MEG data (see “MEG processing and analysis”). MRIs could not be acquired for 3 participants due to dropout and screening contraindications. All imaging analysis was therefore conducted on the remaining 26 datasets.

### MEG acquisition

Data were recorded at 1.2 KHz with an Elekta Neuromag VectorView 306 MEG system with 102 magnetometers and 102 pairs of orthogonal planar gradiometers. Head position indicator (HPI) coils were placed at 4 locations on the head to record head position relative to the MEG sensors at the start of each run. Head landmarks (preauricular points and nasion) and 200 points on the scalp, face, and nose were digitised using a Polhemus Isotrack II system. EEG electrodes were placed above and below the left eye and on the temples to record horizontal and vertical EOG, and on each wrist to record ECG.

### Rapid frequency tagging

During presentation of the dot-motion stimulus, the 2 saccade targets flickered rapidly at 41 Hz and 45 Hz (counterbalanced across participants). The targets flickered sinusoidally between black and white (greyscale) at a refresh rate of 1.44 KHz. To achieve this high rate of presentation, we used a PROPixx DLP LED projector (VPIxx Technologies, Saint-Bruno-de-Montarville, Canada). In postexperiment debriefing, no participant reported awareness of the flicker.

### MEG processing and analysis

MEG analysis was performed using fieldtrip [64], OSL (https://github.com/OHBA-analysis/osl-core), and custom MATLAB scripts.

Data were first Maxwell filtered using the MaxFilter program (Elekta Instrument AB, Stockholm, Sweden); Maxfilter is a method for separating parts of the recorded MEG signal that arise from external noise and neuronal activity, respectively. MRI and MEG data were coregistered using RHINO (Registration of Headshapes Including Nose in OSL). Data were downsampled to 200 Hz, bandpass filtered between 1 and 80 Hz, and bandstop filtered around the line-noise frequency of 50 Hz. Trials containing outlier values were automatically detected and removed using function osl_detect_artefacts with default settings, and independent component analysis was used to automatically remove additional artefacts associated with eye blinks, ECG, and line noise.

A single-shell forward model was constructed from each participant’s anatomical MRI. Sensor data were projected onto an 8-mm grid using an LCMV vector beamformer [41,65] carried out on each 20-minute MEG run separately. The grid was constructed using a template (MNI152) brain, then each participants’ anatomical MRI was warped to the template brain and the inverse warp applied to the grid. This ensures comparability of source reconstructions across participants. Because MaxFilter considerably reduces the dimensionality of the data—to approximately 64—the data covariance matrix was reduced to 50 dimensions using PCA. Eigenvalue decomposition of magnetometer and gradiometer channels was performed in order to normalise each sensor type to ensure that both sensor types contributed equally to the covariance matrix calculation [66].

### Parietal cortex and frontal eye field low-frequency ROI analysis

The parietal ROI was defined with respect to both anatomy (the Harvard Oxford cortical atlas) and function (band-limited power at the specific frequency-tag frequencies)—see “Frequency tagging analysis.” This was used to construct “virtual channels” for left and right FEF based on a symmetric orthogonalisation method [67].

FEF was defined with reference to a probabilistic atlas of human visual and oculomotor cortical regions [68].

A limitation of beamforming is that the polarity of the recovered signal cannot be disambiguated; source activity is inherently *sign-ambiguous*. This means that conventional event-related averaging in the time domain is problematic. Accordingly, we performed time-frequency analysis and extracted time-varying power in the low frequency range (2.8 to 8.4 Hz) as a proxy for the event-related field or ERF, the magnetic field arising from local field potentials in cortex [42], as power is not sign-ambiguous but rather always positive. Oscillatory power was then computed via time-frequency analysis using a 500-ms sliding window multiplied with a Hanning taper, at 0.8 Hz frequency resolution. We then averaged across the low-frequency 2.8 to 8.4 Hz band as a proxy for evoked activity, resulting in an ROI-specific single-trial time series. We focussed on low frequencies rather than higher frequencies because the network model is dominated by low-frequency responses and does not exhibit higher frequency oscillations [34].

Inspection of the MEG data revealed 2 evoked responses with comparable durations; one 100 to 500 ms following the onset of the incoherent motion, and one 100 to 500 ms following the onset of coherent motion. To visualise the effects of task variables on these evoked responses, we took the average low-frequency power across each time window for each level of coherence (10%, 30%, 50%, 70%, and 90% of dots moved either left or right, Fig 1E and 1F), competition (50%, 70%, or 90% of coherently moving dots moved in the same direction, Fig 1H and 1I), and prior strength (unsigned value output from Bayesian model *abs*(*E*(*q*_*t*_) − 0.5), S2 Fig). Because the latter is a continuous variable, we performed a tertile split into “weak” (p(right) close to 0.5), “medium,” and “strong” (p(right) close to 0 or 1) values for visualisation purposes.

To determine the effect of stimulus variables (coherence competition) and prior belief strength on low-frequency power in the FEF and parietal cortex, we applied linear regressions with these 3 predictor variables in 2 ways. Firstly, for visualisation, at every time point (Figs 3E, 3H, 3K, S4A, S4C and S4E). Secondly, for statistical purposes, over the average power values in a preselected box from 100 to 500 ms after coherent motion onset (Figs 3F, 3I, 3L, S4B, S4D and S4F), based on the results from the neural mean field model (Fig 3D, 3E and 3J).

### Frequency tagging analysis

To analyse the effect of the rapidly flickering saccade targets on posterior brain regions, we used a combination of anatomical and data-driven selection criteria to focus on the brain regions that produced the strongest tagging response. We selected a 3-second time window from 800 ms before the onset of coherent motion to 2.2 seconds afterwards—i.e., almost the entire period the targets were flickering—and calculated the Fourier transform at all voxels for all participants and averaged across all artefact-free trials. We then compared power at the left-target frequency (41 Hz for half the participants and 45 Hz for the other half) with power at the right-target frequency, which revealed strong effects of tagging in voxels in parietal and occipital regions. We then used an anatomical atlas to create weighted maps defined by the conjunction of statistically significant differences between tag-frequency power and the parietal cortex anatomical label. The left parietal ROI consisted of all voxels in left parietal cortex that showed significantly greater power at the left-hemisphere tagging frequency than the right, and the reverse was true for the right parietal ROI. We multiplied these weight maps with the source-space data to create “virtual channels” in left and right parietal cortices. We then performed time-frequency analysis on each virtual channel at the relevant tag frequency (41 Hz or 45 Hz, depending on the flicker rate of the contralateral frequency tag), using a longer 1,000-ms sliding window to increase frequency resolution. This produced time-resolved estimates of power at the relevant tag frequency in the left and right parietal ROIs.

For the analysis of prior belief (Fig 4C and 4D), we expected effects prior to coherent motion onset and, therefore, focused on the 1-second epoch of incoherent motion, focusing on a time window 500 to 100 ms before coherent motion onset due to low SNR in the first part of the incoherent motion epoch as frequency tagging activity ramped up (Fig 4A).

We performed a tertile split on the prior, but—as we expected *hemispherically lateralised* effects due to the retinotopic organisation of parietal cortex—we used the signed prior, splitting into “strong left,” “weak,” and “strong right.” We then averaged tag power values in the selected time window, at each level of prior and compared these using a 1 × 3 ANOVA with linear contrast. We also conducted a linear regression using the parametric prediction of the prior probability of dots moving right, *E*(*q*_*t*_), as per Eq 5, as explanatory variable, in the same time window. To account for the possibility that frequency tag power would differ substantially between hemispheres due to the different driving frequencies, we applied an interhemispheric correction, whereby for each hemisphere at each time point, the within-hemisphere mean signal was subtracted, and the global mean (average of both hemispheres) was added.

Because we did not have a strong a priori prediction about the time window in which parietal activity might be driven by belief-decision congruence, we used a nonparametric cluster-based permutation test [69] over all time points to determine whether there was a difference between the congruent and incongruent conditions. We again performed a tertile split on the prior; strong left, weak, strong right, and a three-way split on the dot directions; mostly left motion (10% or 30% right), equal motion (50% right), or mostly right motion (70% or 90% right). We then defined trials where the dominant motion direction and prior agreed as “congruent,” and trials where they were opposite as “incongruent,” and performed the cluster-based permutation test on the participant conditional means.

### Time course analysis

To illustrate how the computational components of active sampling evolve in time, we calculated, for every time point in the task epoch (from the onset of incoherent motion until the cessation of coherent motion), repeated measures ANOVAs comparing relevant task variables. We compared, respectively, frequency tagging activity in parietal cortex as a function of prior strength (strong contralateral, weak, strong ipsilateral), low-frequency activity in FEF and parietal cortex as a function of stimulus properties (coherence; 10%, 30%, 50%, 70%, 90%, competition; low, medium, high), and parietal frequency tagging activity as a function of prior/stimulus congruence (congruent, incongruent). To illustrate the temporal cascade of events, we found the time point at which each F-statistic was maximal.

## Supporting information

S1 Fig Lagged logistic regression reveals integration kernels over previous trials. Lagged logistic regression of current and previous task variables on current choice. Error bars indicate standard error of the mean across participants.
(EPS)

S2 Fig No evidence that prior belief strength modulates low-frequency activity in frontal or parietal cortex. Signals related to strength of prior belief. All plotting conventions as Fig 3, main text. (**A**) Neural mean-field model predicts a parametric increase in activity as a function of strength of prior belief. (**B**, **C**) FEF low-frequency power. (**D**, **E**) Parietal cortex low-frequency power. (**F**, **G**) Results from general linear model. The data underlying this figure can be found in Zenodo (DOI: 10.5281/zenodo.10209832).
(EPS)

S3 Fig MEG responses to stimulus variables (coherence and competition): Expanded to show additional detail. (**A**) MEG response to dot-motion stimulus, as a function of stimulus coherence. Plotting conventions identical to Fig 3E, except for y-axis, which has been expanded to visualise the parietal response to the incoherent motion onset at time −1. (**B**) As (**A**), but as a function of stimulus competition, identical to Fig 3H. (**C**) Plots of individual participants MEG low-frequency power values as a function of stimulus coherence. Plotting conventions are identical to Fig 3F, except that lines have been added to connect individual participants’ data points so that individual slopes can be observed. Bars indicate group means. Thick black lines indicate group medians. (**D**) As (**C**), but as a function of stimulus competition.
(EPS)

S4 Fig Stimulus properties parametrically modulate low-frequency activity in FEF similarly to parietal cortex. As Fig 3, but plotting the effects of stimulus coherence (**A**, **B**) and competition (**C**, **D**) on low-frequency activity in the FEF. (**E**, **F**) GLM analysis confirms significant effects of both variables in the 100–500 ms following stimulus onset. The data underlying this figure can be found in Zenodo (DOI: 10.5281/zenodo.10209832).
(EPS)

S5 Fig Effects of stimulus variables (coherence, competition) on frequency-tagging activity in parietal cortex. Analysis of parietal cortex frequency tagging data analogous to low-frequencies (Fig 3). (**A**) Parietal frequency tagging time series as a function of stimulus coherence. (**B**) Averaged across 100–500 ms time window, dashed box in (**A**). (**C**, **D**) As (**A**, **B**), but as a function of stimulus competition. (**E**, **F**) As (**A**, **B**), but as a function of “signed competition,” i.e., proportion of coherent motion moving contralateral to ROI. The data underlying this figure can be found in Zenodo (DOI: 10.5281/zenodo.10209832).
(EPS)

S6 Fig Ability to detect lateralised task processing in FEFs and parietal cortex—Dot motion. (**A**) Simulated synaptic inputs for right-motion accumulator of neural mean-field model, as a function of percentage coherent dots moving right. (**B**) As (**A**), but left-motion accumulator. (**C**) Source-reconstructed activity from left FEF, same colour convention. (**D**) Individual participant data in stimulus-evoked window. (**E**, **F**) As (**C**, **D**), but right FEF. (**G**-**J**) As (**C**-**F**), but parietal cortex. The data underlying this figure can be found in Zenodo (DOI: 10.5281/zenodo.10209832).
(EPS)

S7 Fig Ability to detect lateralised task processing in FEFs and parietal cortex—Prior belief. S6 Fig: (**A**) Simulated synaptic inputs for right-motion accumulator of neural mean-field model as a function of prior belief. (**B**) Source reconstructed activity in left FEF. (**C**) Individual participant data in stimulus-evoked window. (**D**-**F**) As (**A**-**C**), but left-motion accumulator and right FEF. The data underlying this figure can be found in Zenodo (DOI: 10.5281/zenodo.10209832).
(EPS)

S8 Fig Model 2—Parameter fitting. Model log likelihood for each combination of parameters (W, θ) for each participant. Colours represent model log likelihood (arbitrarily scaled). The maximum likelihood values of (W, θ) were used in Model 2 as described in methods above.
(EPS)

## Abbreviations

CPP: centro-parietal positivity
ERF: event-related field
FEF: frontal eye field
LCMV: linearly constrained minimum variance
LIP: lateral intraparietal area
MEG: magnetoencephalography
PSE: point of subjective equality
RDK: random dot kinematogram
ROI: region of interest
